# The Application of Small Molecules to the Control of Typical Species Associated With Oral Infectious Diseases

**DOI:** 10.3389/fcimb.2022.816386

**Published:** 2022-02-21

**Authors:** Sirui Yang, Xiaoying Lyu, Jin Zhang, Yusen Shui, Ran Yang, Xin Xu

**Affiliations:** ^1^State Key Laboratory of Oral Diseases, National Clinical Research Center for Oral Diseases, West China Hospital of Stomatology, Sichuan University, Chengdu, China; ^2^Department of Cariology and Endodontics, West China Hospital of Stomatology, Sichuan University, Chengdu, China; ^3^Department of Pediatric Dentistry, West China Hospital of Stomatology, Sichuan University, Chengdu, China

**Keywords:** oral microbiota, small molecules, antimicrobial agents, dental plaque biofilm, dental caries, periodontal diseases

## Abstract

Oral microbial dysbiosis is the major causative factor for common oral infectious diseases including dental caries and periodontal diseases. Interventions that can lessen the microbial virulence and reconstitute microbial ecology have drawn increasing attention in the development of novel therapeutics for oral diseases. Antimicrobial small molecules are a series of natural or synthetic bioactive compounds that have shown inhibitory effect on oral microbiota associated with oral infectious diseases. Novel small molecules, which can either selectively inhibit keystone microbes that drive dysbiosis of oral microbiota or inhibit the key virulence of the microbial community without necessarily killing the microbes, are promising for the ecological management of oral diseases. Here we discussed the research progress in the development of antimicrobial small molecules and delivery systems, with a particular focus on their antimicrobial activity against typical species associated with oral infectious diseases and the underlying mechanisms.

## Introduction

The oral microbiota, including more than 700 microbial species, are the most complicated microbial communities in human body (Dewhirst et al., 2010). According to the ecological plaque hypothesis, oral microbial dysbiosis leads to the occurrence of oral infectious diseases including dental caries and periodontal diseases, which seriously endanger oral and general health (Theilade, 1986).

*Streptococcus mutans* is well recognized as the major cariogenic species due to its capability of adhesion to tooth surfaces, generation of acid through sugar fermentation, and tolerance and persistence in acidic microenvironment (Hamada et al., 1984; Banas, 2004; Bowen et al., 2018). Currently, the homeostasis between pathogenic and commensal bacteria has attracted increasing attention in the etiology and pathogenesis of dental caries. Dental caries is believed to be initiated by the imbalanced microecology and the overgrowth of acidogenic/aciduric bacteria such as *S. mutans* (Tanner et al., 2016; Samaranayake and Matsubara, 2017). In addition to caries,the microbiological etiology of periodontitis has also been indicated in recent years (Riep et al., 2009). *Porphyromonas gingivalis, Tannerella forsythia*, and *Treponema denticola*, commonly known as the “red complex”, are well recognized as the principal pathogens associated with periodontal destruction (Socransky et al., 1998). Currently, periodontitis is believed to be the consequence of a broadly-based dysbiotic alteration in periodontal microbiota, whereby some keystone species such as *P. gingivalis* triggers the development of this disease (Hajishengallis and Lamont, 2012). *Candida albicans* is a commensal fungal species colonizing human oral mucosal surfaces. In the immunocompromised individuals, *C. albicans* becomes opportunistic pathogen causing mucosal and disseminated infections (Metwalli et al., 2013). Intriguingly, *C. albicans* robustly interacts with oral bacteria, and this cross-kingdom interaction enhances the virulence of both fungi and bacteria, and ultimately aggravates oral diseases (Dewhirst et al., 2010; Peters et al., 2012). It has been proven that *C. albicans* is closely involved in the occurrence of various oral diseases including early childhood caries, root caries, periodontitis, endodontic infections, oral mucositis and facial space infections (Krom et al., 2014).

As an adequate plaque control by mechanical means such as brushing and flossing is difficult to achieve by most patients, mouth rinses containing antimicrobial agents are considered as an effective adjuvant measure to control dental caries (Lim and Kam, 2008; Rath and Singh, 2013). Chlorhexidine (CHX) is widely used to control oral pathogens due to its robust antimicrobial activity and broad spectrum (Jones, 1997). However, CHX has drawbacks such as taste confusions, mucosal soreness, oral microbial dysbiosis and drug resistance, which limit its long-term application (Jones, 1997; Walsh et al., 2015). Bacterial drug resistance is one of the main threats to human health (Kumar and Balbach, 2017), limiting the options of clinical treatment for oral infectious diseases (Hegstad et al., 2010). Long-term use of CHX could cause microbial resistance in microbes, including *Staphylococcus aureus, Enterococcus faecalis* and *Klebsiella pneumoniae* (Wang et al., 2017). Therefore, novel agents are urgently needed to control oral infectious diseases. Antimicrobial small molecules are a series of natural or synthetic bioactive compounds showing good antimicrobial activity against microbiota associated with infectious diseases (Worthington et al., 2012). Small molecules can be developed *via* various approaches. Drug repurposing, drug screening from existing small-molecule libraries or natural resources, and target-based designing are most common approaches to the development of small molecules that target oral microbiota and consequently benefit oral infectious disease control. In this review, we aimed to discuss the research progress in the development of antimicrobial small molecules and delivery systems, with a particular focus on: 1) their antimicrobial activity against keystone bacteria including *S. mutans*, *P. gingivalis* and *C. albicans*; 2) their inhibitory effects on the pheromones that mediate interspecies communications within polymicrobial communities; 3) the research progress in the development of delivery systems that enhance the antimicrobial activity of small molecules in the management of oral infectious diseases.

## Small Molecules That Inhibit Keystone Bacteria Associated With Oral Infectious Diseases

### Streptococcus mutans

*S. mutans* is generally recognized as the key cariogenic species, particularly due to its capability of driving the shift of oral microbiota towards a more acidogenic/aciduric community that ultimately causes tooth demineralization and visible decay (Marsh, 2010).

Different small molecular antibiotics from natural products and synthetic compounds have been identified against *S. mutans*. Drug-repositioning is a commonly used approach to the identification of antimicrobial agents that inhibit *S. mutan*s. Nitrofuran, with a mode of action similar to that of nitroimidazole, shows inhibitory activity on oral bacteria such a*s S. mutans* and *Enterococcus faecalis* (Silva et al., 2014; Ang et al., 2017). Based on the antimicrobial activity of nitrofuran, our group synthesized and identified a compound named ZY354, a water-soluble hybrid of indolin-2-one and nitrofuran, which showed potent antimicrobial activity and selectivity against *S. mutans* compared with CHX (Zhang et al., 2019). Saputo et al. (2018) screened and identified 126 FDA-approved small molecules that exhibited antimicrobial activity against planktonic growth of *S. mutans*, among which 24 drugs inhibited biofilm formation, 6 drugs killed pre-existing biofilms, and 84 drugs exhibited both bacteriostatic and bactericidal effects against *S. mutans* biofilms. Napabucasin (NAP) is a phase III clinical trials anticancer drug with antibacterial activity against *Escherichia coli*, *Streptococcus faecalis*, and *Staphylococcus aureus* (Kuete et al., 2007; Kuete et al., 2011). Our group repurposed NAP against oral *streptococci* and found good antimicrobial activity of NAP against *S. mutans* biofilms (Kuang et al., 2020). Besides, NAP showed relatively lower antibacterial effect on oral streptococci than CHX with mild cytotoxicity on oral cells. We further redesigned and synthesized a novel small molecule based on NAP, namely LCG-N25, which exhibited potent antibacterial activity, lessened cytotoxicity, and induced no drug resistance of cariogenic *S. mutans* (Lyu et al., 2021a). Chen et al. screened approximately 2600 compounds and identified an antagonist of calcium-sensing receptor, namely NPS-2143, which exhibited antimicrobial activity against methicillin-resistant *S. aureus* (MRSA) (Chen Y et al., 2019). Further modifications of NPS-2143 yield a compound II-6s (Chen Y et al., 2019). Our group demonstrated that II-6s effectively inhibited the growth of *S. mutans*, reduced EPS production and induced no drug resistance in *S. mutans* after repeated treatment as compared to CHX, indicating its potential use in the control of dental caries (Zhang J et al., 2021).

Phenotypic screening is also a reliable approach to the identification of new antimicrobials. Antigen I/II, also known as Pac, mediates the sucrose-independent adhesion of *S. mutans* (Munro et al., 1993; Jenkinson and Demuth, 1997; Love et al., 1997), while Gtfs (GtfB, GtfC, and GtfD) mediate its sucrose-dependent adhesion (Bramstedt, 1968) and play an important role in the interspecies coaggregation and the development of oral biofilms (Bowen and Koo, 2011; Kim D et al., 2020). Rivera-Quiroga et al. performed a high-throughput screening of 883551 molecules, and identified three molecules, namely ZINC19835187 (ZI-187), ZINC19924939 (ZI-939) and ZINC 19924906 (ZI-906), which targeted antigen I/II and inhibited the adhesion of *S. mutans* with low cytotoxicity (Rivera-Quiroga et al., 2020). Wu et al. also screened and identified a molecule called 2A4, showing selectivity on *S. mutans* in multispecies biofilms *via* inhibiting antigens I/II and Gtfs (Liu et al., 2011). The same group also performed a structure-based virtual screening of 500,000 compounds against the GtfC catalytic domain and identified a lead compound, namely G43, which selectively bond Gtfc and significantly inhibited the biofilm formation and cariogenicity of *S. mutans* (Zhang et al., 2017). They further synthesized an analog of G43, named III_F1_, which remarkably reduced dental caries in rats (Nijampatnam et al., 2021). Ren et al. screened 15000 molecules based on the structure of GtfC protein domain and identified a quinoxaline derivative, 2-(4-methoxyphenyl)-N-(3-{[2-(4-methoxyphenyl)ethyl]imino}-1,4-dihydro-2-quinoxalinylidene)ethanamine, which selectively bond GtfC, reduced the synthesis of insoluble glucans, inhibited *S. mutans* biofilm and reduced caries in rats (Ren et al., 2016). SrtA is membraned-bond transpeptidase that catalyzes surface protein antigen I/II, thus contributing to the biofilm formation of *S. mutans* (Lee and Boran, 2003; Krzysciak et al., 2014; Chen X et al., 2019; Wang et al., 2019). Recently, several SrtA inhibitors have been identified from either natural products or synthetic compounds (Park W et al., 2017; Song et al., 2017). Samanli et al. screened and identified a SrtA inhibitor, namely CHEMBL243796 (kurarinone), which showed better affinity to SrtA as compared to CHX (Salmanli et al., 2021). In addition to the aforementioned molecules that have been proven to inhibit antigen I/II and Gtfs, several synthetic molecules have been designed and showed antibacterial effects against *S. mutans.* Kim et al. synthesized a series of pyrimidinone or pyrimidindione-fused1,4-naphthoquinones with antibacterial effects *via* pharmacophore hybridization, and some derivatives exhibited notable bacteriostatic and bactericidal effects against *S. mutans* in both resistant and sensitive strains (Kim K et al., 2020). Zhang et al. screened 100 trimetrexate (TMQ) analogs and identified 3 compounds with good selectivity against *S. mutans* (Zhang et al., 2015). Garcia et al. screened a series of 2-Aminoimidazole (2-AI) derivatives, and identified a small molecule 3F1, which specifically disturbed *S. mutans* biofilms and reduced caries in rats (Garcia et al., 2017). Besingi et al. screened and identified a benzoquinone derivative AA-861, which exhibited antibiofilm effects against *S. mutans* by targeting amyloid fibrils, an important scaffold in *S. mutans* biofilms (Besingi et al., 2017). Chen et al. also screened and identified a small molecule, namely D25, which targeted amyloid fibrils and selectively inhibited *S. mutans* biofilms (Chen et al., 2021).

Natural products and their derivatives also accounted for a large number of antimicrobial small molecules due to their structural diversity and biological activity (Davison and Brimble, 2019; Newman and Cragg, 2020). The tea polyphenols epigallocatechin gallate (EGCG) has been identified to inhibit *S. mutans* for decades. EGCG not only inhibits planktonic bacteria but also reduces the biofilm formation of *S. mutans* by inhibiting Gtfs. In addition, EGCG can inhibit lactate dehydrogenase and F_1_F_0_-ATPase, and thus reduces the acidogenicity and aciduricity of *S. mutans* (Xu et al., 2011; Xu et al., 2012; Hairul Islam et al., 2020). A lipid-soluble green tea polyphenols which is designed based on EGCG, namely epigallocatechin-3-gallate-stearate (EGCG-S), shows an increased stability and antibiofilm activity comparable to CHX (Melok et al., 2018). Moreover, the EGCG is less cytotoxic compared with CHX, and shows anti-inflammatory effects on *S. mutans*-stimulated odontoblast-like cells (Stavroullakis et al., 2021), indicating a good prospect in the management of oral infectious diseases. Propolis and its derivatives such as apigenin and trans-trans farnesol (tt-farnesol) have been identified to show a good antimicrobial activity against *S. mutans* and exhibit notable biological activities against dental caries for decades (Koo et al., 2002; Koo et al., 2003; Cardoso et al., 2010; Veloz et al., 2016). Apigenin has been shown to inhibit Gtfs, specifically GtfB and GtfC. tt-farnesol shows anti-caries effects by reducing cell viability and destabilizing oral biofilms rather than affecting Gtfs activities (Koo et al., 2002; Koo et al., 2005; Jeon et al., 2011). Caffeic acid phenethyl ester (CAPE), another extracted compound from propolis, shows broad-spectrum antimicrobial activity against various microbes including *Enterococcus faecalis*, *S. aureus*, *Bacillus subtilis*, *Pseudomonas aeruginosa*, etc (Velazquez et al., 2007). Niu et al. showed that CAPE affected the morphology of *S. mutans* biofilms, inhibited biofilm formation and maturation and reduced EPS production (Veloz et al., 2019; Niu et al., 2020). In addition, plenty of other natural compounds have also been identified exhibiting antibacterial effects against *S. mutans.* Piceatannol, a plant-derived stilbene, can target GtfC domain and inhibit glucans production, and thus reduces *S. mutans* biofilms formation (Nijampatnam et al., 2018). Piceatannol can also inhibit F_1_F_0_-ATPase of *S. mutans*, and thus suppresses the aciduricity of S. mutans (Sekiya et al., 2019). In addition, curcumin, a phytopolyphenols from traditional medicine known as turmeric, and its analog desmethoxycurcumin (DMC), also show inhibitory effect on F_1_F_0_-ATPase of *S. mutans* and thus reduce its growth in acidic conditions (Sekiya et al., 2014; Nakanishi-Matsui et al., 2016; Sekiya et al., 2019). Ursolic acid, a plant-derived compound, shows inhibitory effects on EPS synthesis and biofilm formation of *S. mutans* (Kim et al., 2013; Lyu et al., 2021b). Astilbin, a flavanone compound from *Rhizoma Smilacis Glabrae* and β-sitosterol from kemangi, can inhibit SrtA activity and thus reduces the biofilm formation of *S. mutans* (Wang et al., 2019; Evangelina et al., 2021).

Small molecules designed for specific target is another approach to the inhibition of *S. mutans.* Charles et al. synthesized several peptides spanning residues 803-185 of antigen I/II, and identified a synthetic peptide p1025 that inhibited antigen I/II binding to salivary receptors by forming adhesion epitopes in a dose-dependent way. The effect of p1025 against *S. mutans* was relatively stable, and it was able to selectively inhibit *S. mutans* recolonization to tooth surface (Kelly et al., 1999; Younson and Kelly, 2004; Li et al., 2009). Small molecules that show inhibitory effects on *S. mutans* are summarized in **Table 1**.

**Table 1:** Small molecules that inhibit *S. mutans*.

Small molecules	Chemical structure	Mechanisms	Antimicrobial activity	Reference
Drug-repositioning				
LCG-N25	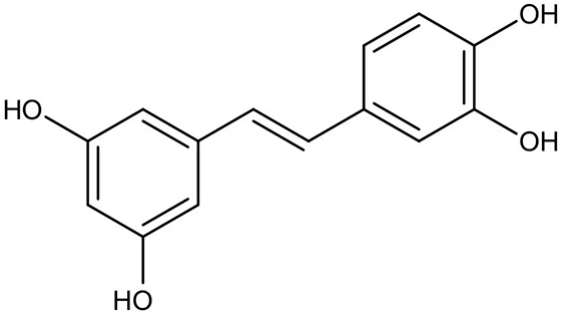	Inhibit both the planktonic cells and biofilms formation of *S. mutans*	MIC_90_: 0.5 μg/ml	(Lyu et al., 2021a)
MBC_90_: 15.6 μg/ml				
Napabucasin	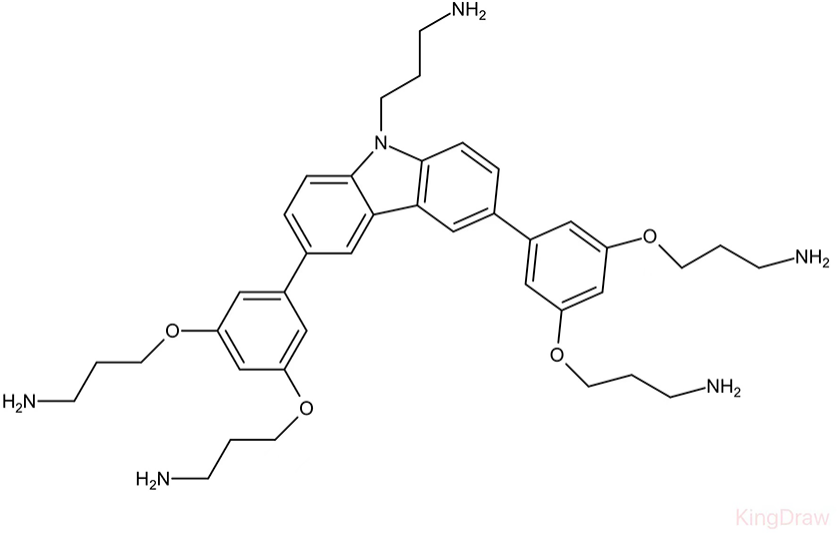	Inhibit *S. mutans* biofilms	MIC_90_: 3.91 μg/ml	(Kuang et al., 2020)
			MBC_90_: 15.63 μg/ml	
			MBIC_90_: 1.95 μg/ml	
			MBRC_90_:62.5μg/ml	
ZY354	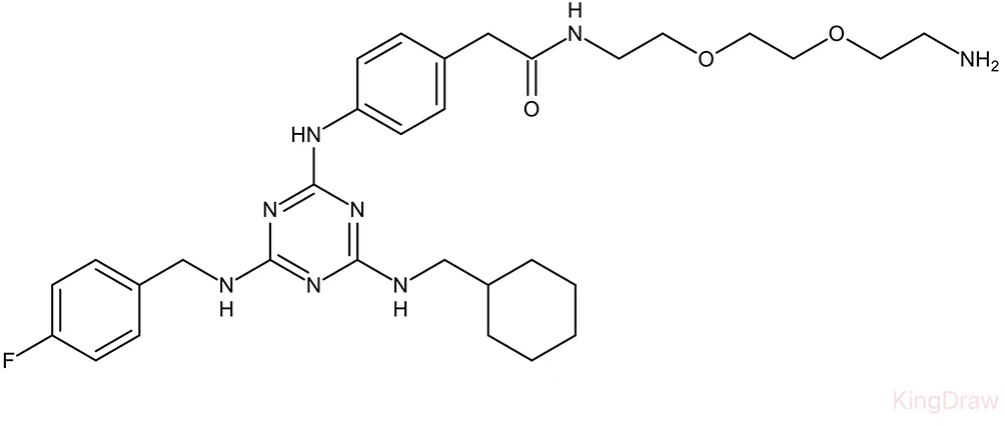	Inhibit *S. mutans* growth and selectively inhibit the biofilm formation of *S. mutans*	MIC_90_: 0.24 μg/ml	(Zhang et al., 2019)
			MBC_90_: 1.95 μg/ml	
			MBIC_90_: 0.24 μg/ml	
			MBRC_90_: 31.25μg/ml	
II-6s	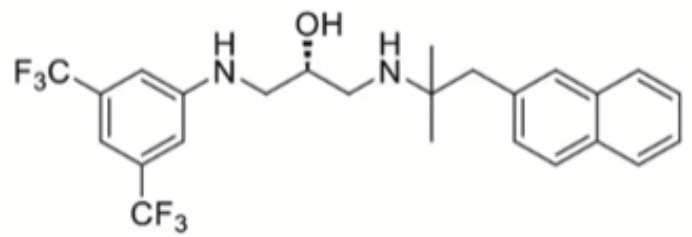	Inhibit growth and exopolysaccharides (EPS) generation of *S. mutans*;	MIC_90_: 3.91 μg/ml	(Zhang J et al., 2021)
			MBC_90_: 15.63 μg/ml	
			MBIC_90_: 3.91 μg/ml	
			MBRC_90_: 62.5 μg/ml	
		inhibit the demineralization of tooth enamel and induce no drug resistance in *S. mutans*		
Phenotypic screening from libraries				
Compound 3F1	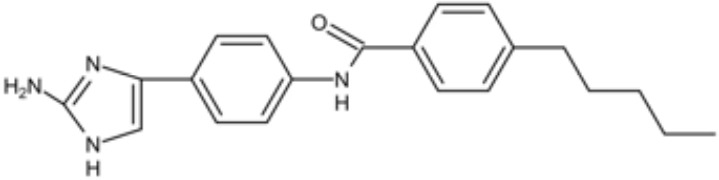	Specifically disturb *S. mutans* biofilms in a mixed biofilm	MDC: 5 µM	(Garcia et al., 2017)
D25		Selectively inhibit S. mutans biofilms without interfering planktonic cells	Inhibit *S. mutans* biofilms at the concentration of 3.125–25 μg/mL	(Chen et al., 2021)
G43	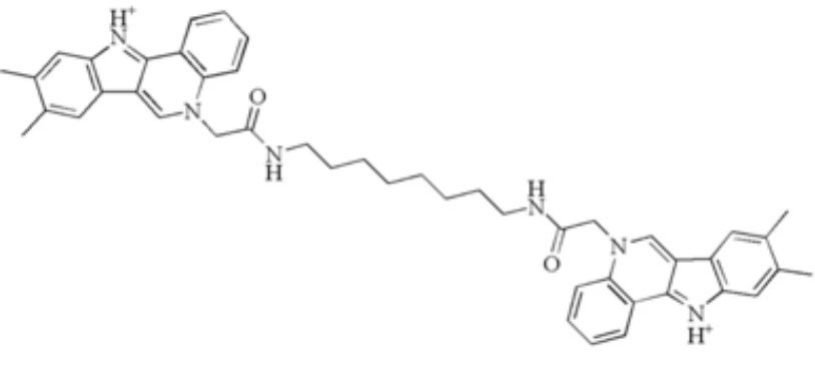	Inhibit S. mutans biofilm formation by selectively binding to GtfC	Inhibit more than 85% of *S. mutans* biofilms at 12.5 μM	(Zhang et al., 2017)
Pyrimidinone or pyrimidindione-fused 1,4-naphthoquinones	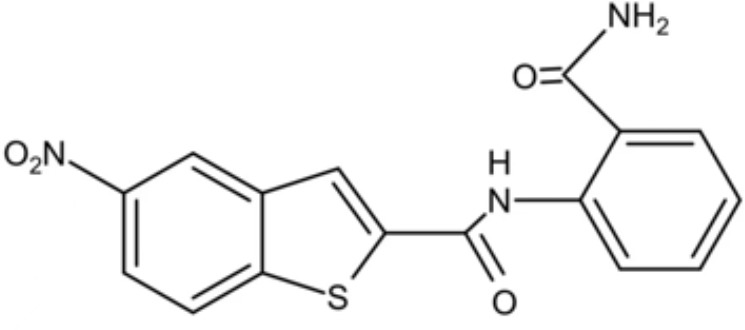	Show bacteriostatic and bactericidal effects against		(Kim K et al., 2020)
		*S. mutans* in both resistant and sensitive strains		
ZINC19835187 (ZI-187)	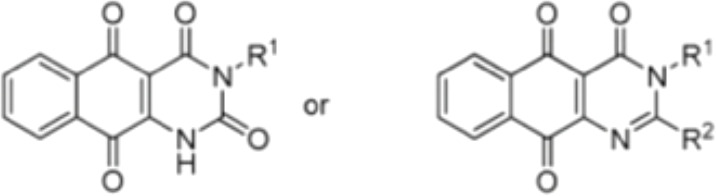	Inhibit *S. mutans* adhesion and biofilm formation by targeting antigens I/II	Show no inhibitory effects on *S. mutans* growth at 10-100-1000 μM	(Rivera-Quiroga et al., 2020)
ZINC19924939 (ZI-939)	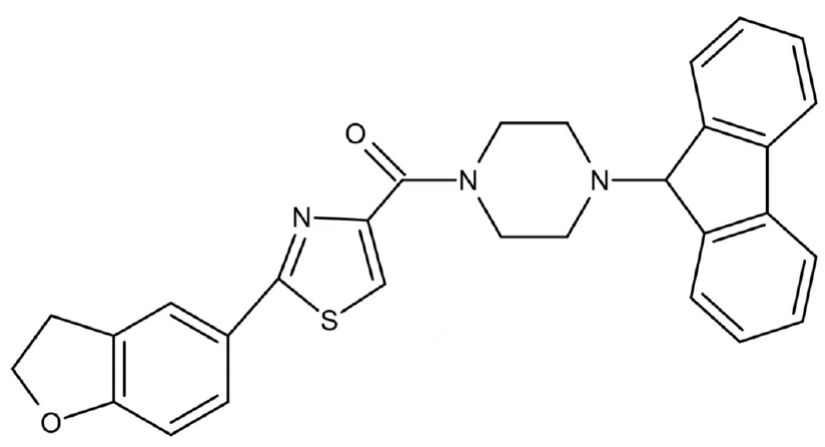			
ZINC 19924906 (ZI-906)	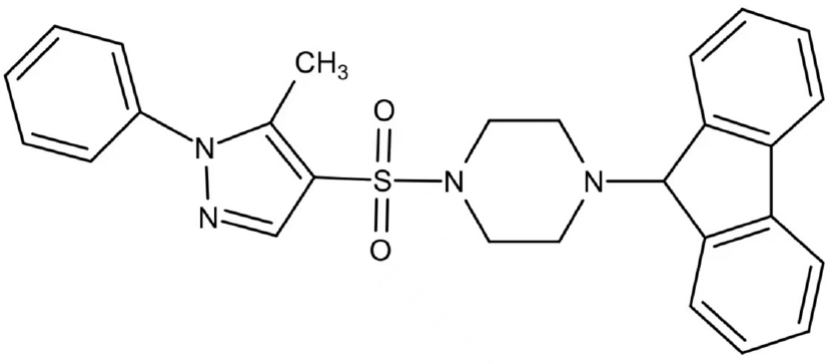		Show inhibitory effects on adhesion than 90% at 200 μM (ZI-187 at 100 μM)	
2A4	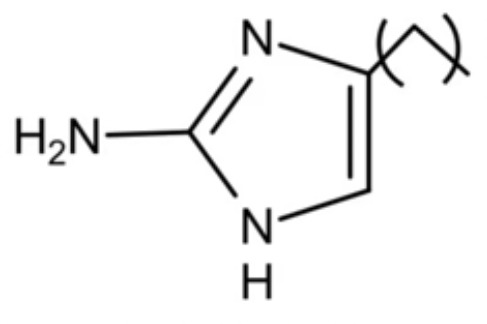	Inhibit *S. mutans* adhesion and biofilm formation by targeting antigens I/II and glucosyltransferases (Gtfs)	MIC50: 2.0 ± 0.5 μM	(Liu et al., 2011)
			MBIC50: 0.94 ± 0.02 μM.	
2-(4-methoxyphenyl)-N-(3-{[2-(4-methoxyphenyl)ethyl]imino}-1,4-dihydro-2-quinoxalinylidene) ethanamine	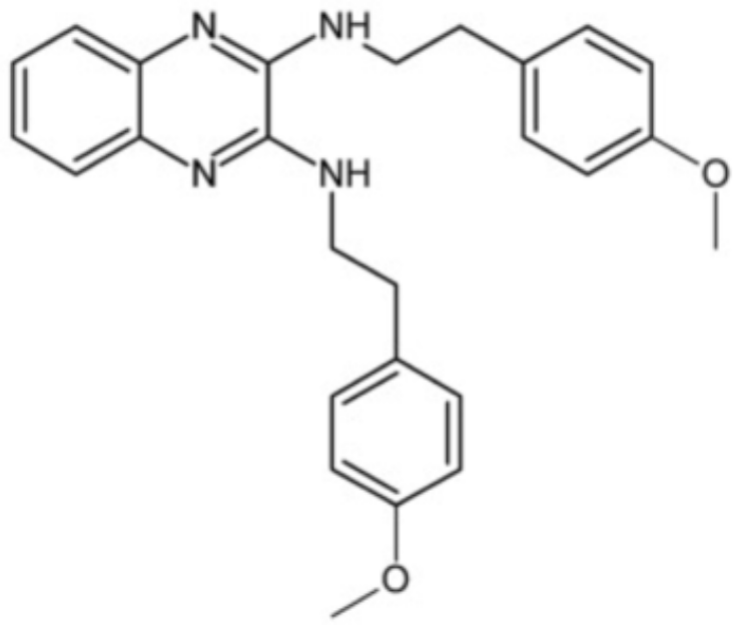	Inhibit the biofilm formation and destroy mature biofilms without killing *S. mutans* by inhibiting GtfC	Reduce 79% *S. mutans* biofilms cell viable count at 10 μg/ml	(Ren et al., 2016)
Natural products screening				
Apigenin	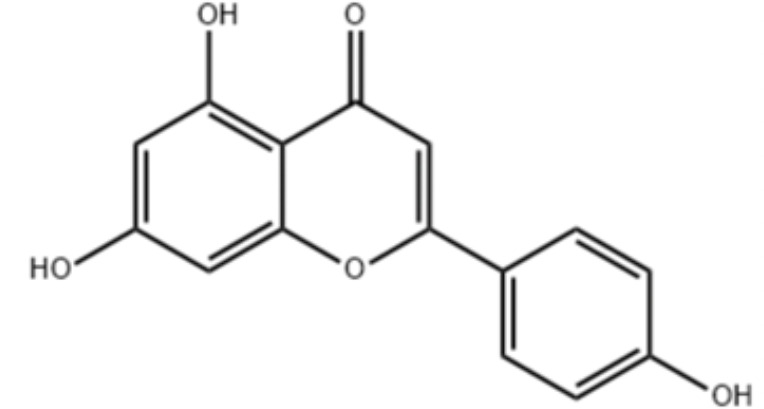	Inhibit Gtfs, specifically GtfB and GtfC;		(Koo et al., 2002; Koo et al., 2005)
β-sitosterol from Kemangi	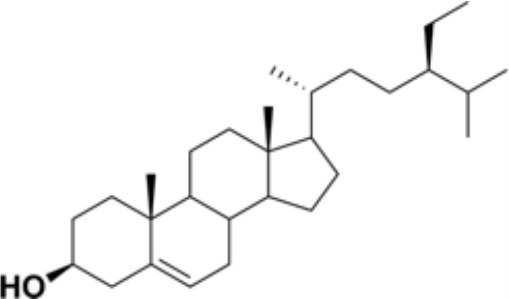	Inhibit *S. mutans* biofilm formation by inhibiting SrtA	MIC_90_: 25000 ppm	(Evangelina et al., 2021)
			MBC_90_: 50000 ppm	
Caffeic acid phenethyl ester	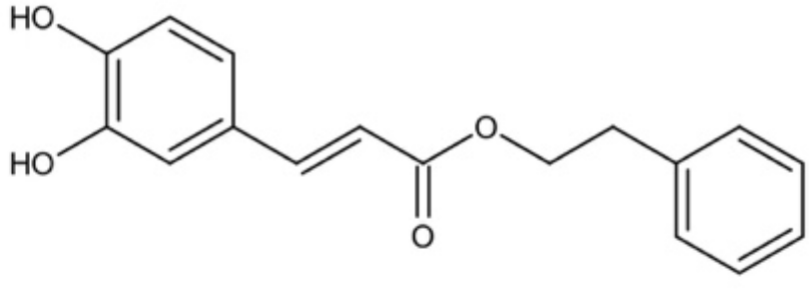	Affect the thickness of S. mutans biofilms	MIC_90_: 80 μg/ml	(Velazquez et al., 2007; Veloz et al., 2019; Niu et al., 2020)
		Inhibit biofilm formation and maturation by reducing EPS production	MBC_90_: 320 μg/ml	
			MBIC_90_: 80 μg/ml	
Curcumin	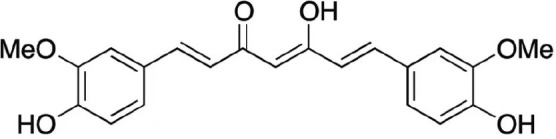	Inhibit F1F0-ATPase and inhibit *S. mutans* growth	MIC_50_: 6 μM	(Nijampatnam et al., 2018; Sekiya et al., 2019)
			Inhibit activity of F1F0-ATPase by 74% at 30μM	
Desmethoxycurcumin	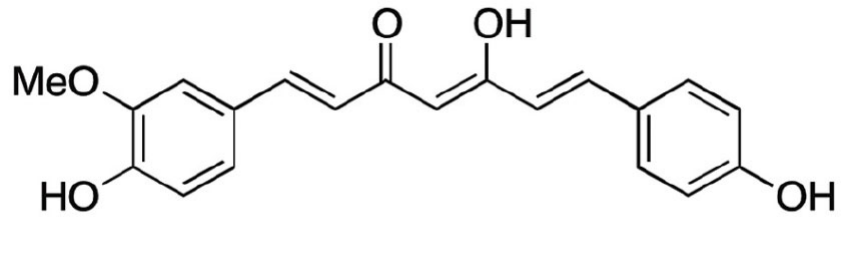		MIC_50_: 4 μM	(Sekiya et al., 2014; Nakanishi-Matsui et al., 2016; Sekiya et al., 2019)
			Inhibit activity of F1F0-ATPase by 82% at 30μM	
Piceatannol	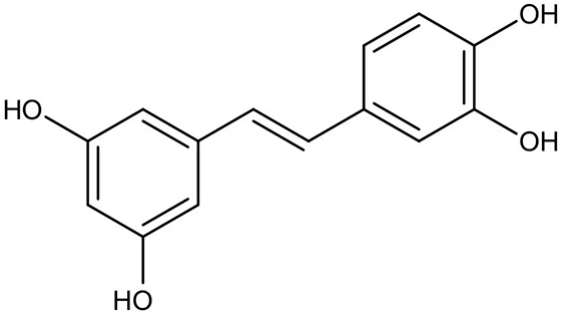		MIC_50_: 39 μM	
			Inhibit activity of F1F0-ATPase by 84% at 200μM	
Epigallocatechin gallate (EGCG)	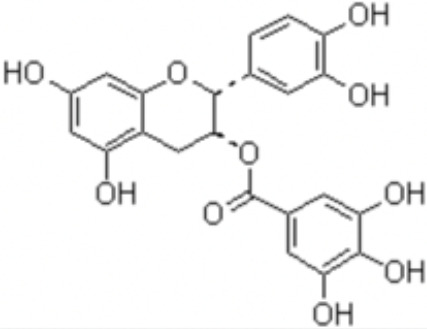	Inhibit *S. mutans* acid production, aciduricity, and biofilm formation	MIC_90_: 15.6 μg/ml	(Xu et al., 2011; Xu et al., 2012; Melok et al., 2018; Hairul Islam et al., 2020; Stavroullakis et al., 2021)
			MBC_90_: 31.25 μg/ml	
Trans-trans farnesol	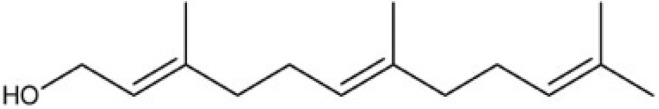	Disrupt membrane integrity, destabilize oral biofilms and reduce the intracellular iodophilic polysaccharides (IPS) accumulation of *S. mutans*	MIC_90_: 125 μM	(Koo et al., 2002; Koo et al., 2005)
		Lipophilic moiety interaction with bacterial membrane	MBC_90_: 500 μM	
Ursolic acid	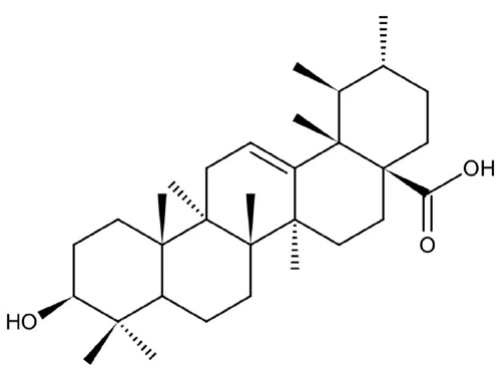	Inhibit biofilm formation and maturation by reducing EPS production	MIC_90_: 7.8 μg/ml	(Kim et al., 2013; Lyu et al., 2021b)
			MBC_90_: 15.6 μg/ml	
Target-base designing				
Compound III _A6_	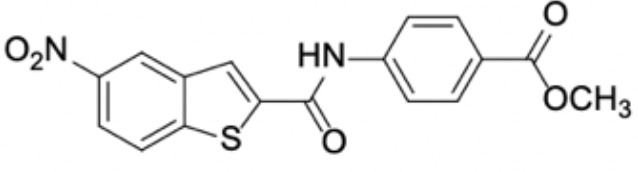	Selectively bond GtfC and significantly inhibit the biofilm formation	MBIC_50_: 9.6 μM	(Nijampatnam et al., 2021)
Compound III _C5_	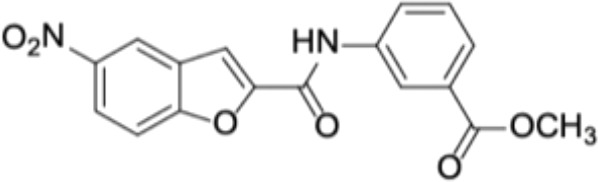		MBIC_50_: 2.7 μM	
Compound III_F1_	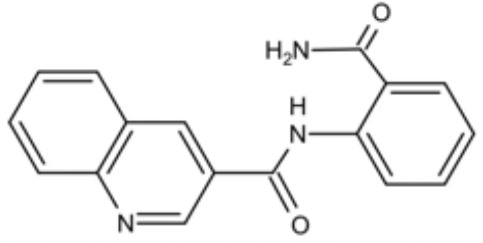		MBIC_50_: 15.3 μM	
Compound III_F2_	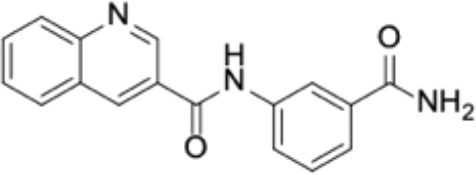		MBIC_50_: 8.6 μM	
P1025		Inhibit the adhesion and biofilm formation of *S. mutans*		(Kelly et al., 1999; Younson and Kelly, 2004; Li et al., 2009)

MIC, minimum inhibitory concentration; MBC, minimum bactericidal concentration; MBIC, minimum biofilm inhibition concentration; MBRC, minimum biofilm reduction concentration; MDC, concentration that a single dose of the small molecule needed to disperse 50% of the biofilm.

### Porphyromonas gingivalis

*P. gingivalis*, despite its relatively low abundance in the periodontal microbiota, has been well recognized as the keystone species of periodontitis (Kojima et al., 1993). *P. gingivalis* expresses Arg-specific cysteine proteinases (gingipains) that help the subversion of recruited leukocytes, leading to uncontrolled overgrowth of other proteolytic and asaccharolytic bacteria in the microbial biofilm, which in turn elevate the complement-dependent destructive inflammation of periodontal tissue and stabilize the transition of periodontal microbiota to a disease-provoking consortium (Hajishengallis et al., 2012; Hajishengallis, 2015). Specific inhibition of *P. gingivalis* could not only suppress its virulence to the periodontium, but also rescue the microbial dysbiosis induced by this keystone pathogen and ultimately shift the microbiota toward a community in favor of periodontal health (Hajishengallis et al., 2012).

The initial colonization of *P. gingivalis* in oral biofilms occurs in the supragingival biofilm (Wright et al., 2013). Adhesion of *P. gingivalis* to streptococci is critical for its pathogenicity. The minor fimbrial antigen (Mfa1) of *P. gingivalis* and streptococcal surface antigen I/II are involved in the interspecies adhension (Park et al., 2005; Daep et al., 2011). Roky identified 3 small molecules, namely N7, N17 and V8 from high-throughput screening of ZINC library, that inhibited *P. gingivalis* adherence to streptococci and reduced its virulence *in vivo*. In addition, compound N17 and V8 showed low cytotoxic activity in both human and marine cells (Roky et al., 2020). Another synthetic compound PCP-III-201, firstly designed as an inhibitor that mimics the natural peptide substrate recognized by Mfa, showed marked inhibition on the adherence of *P. gingivalis* to streptococci by interfering Mfa and antigenI/II interaction, and thus disrupted the formation of mixed biofilms (Tan et al., 2018). 1,2,3-triazole-based peptidomimetics, another natural peptide substrate simulants of Mfa, exhibited inhibitory effect on *P. gingivalis* adherence to oral streptococci by inhibiting the interaction of antigen I/II and Mfa proteins (Patil et al., 2016). Similarly, “the second generation” 1,2,3-triazole-based peptidomimetics based on the first-generation diphenyloxazole were designed and showed inhibitory effect on *P. gingivalis* adherence to *S. gordonii*, indicating the potential use of triazole derivatives in the management of periodontitis (Patil et al., 2019). The major fimbriae of *P. gingivalis* is another adhesin which can bind streptococcal surface component glyceraldehyde-3-phosphate dehydrogenase (GAPDH) and mediates its adhesion to oral streptococci (Maeda et al., 2004; Daep et al., 2006). Three small molecules, namely 2A4, 2D11 and 2E11, which were screened from a library of small molecules based on the 2-aminoimidazole and 2-aminobenzimidazole scaffolds, showed inhibitory effects on *P. gingivalis* by down-regulating Mfa1 and fimA gene expression, thus inhibiting the adherence of *P. gingivalis* to oral streptococci (Wright et al., 2014).

Natural extract is another abundant resource for inhibitors against *P. gingivais* (Abrao et al., 2021). Resveratrol, a natural compound with antimicrobial, antiviral and anticancer activities (Kolouchova et al., 2018), shows inhibitory effects on *P. gingivalis*. The minimum inhibitory concentrations (MIC) of resveratrol against *P. gingivalis* and other clinical strains are in the range of 78.12–156.25 μg/ml. Besides, resveratrol can reduce the *P. gingivalis* biofilm formation and its virulence by downregulating the expression of fimbriae (type II and IV) and proteinases (kgp and rgpA) (Kugaji et al., 2019). A study evaluated a variety of natural products from medicinal plants and identified quite a few small molecular compounds that inhibited the growth and biofilm formation of *P. gingivalis*. Intriguingly, some of the derivatives also inhibited gingipains (Kariu et al., 2017). A natural polyphenol, Quercetin (3,3′,4′,5,7-pentahydroxyflavone), can inhibit gingipains activities and biofilm formation at sub-MIC concentrations. In addition, quercetin down-regulates the expression of virulence-associated genes of *P. gingivalis* (He et al., 2020). Quantum curcumin, a derivative of curcumin, shows notable inhibitory effects on planktonic cells and biofilms of *P. gingivalis*, particularly *via* inhibition of gingipain R and K (Singh et al., 2019).

In addition, several other small molecules have also showed anti-inflammatory effects against periodontitis induced by *P. gingivalis*. Lei et al. synthesized a series of valproic acid pyrazole conjugates, and the most effective molecules 7c not only showed antibacterial effects against *P. gingivalis*, but also reduced inflammation by inhibiting TNF-α, IL-1β and IL-6 (Dong et al., 2021). A sialidase inhibitor, 2-deoxy-2,3-didehydro-N-acetylneuraminic acid (DANA), showed effects on reducing pathogenicity of *P. gingivalis* and exhibited anti-inflammatory prospect (Yu et al., 2021). Small molecules that inhibit *P. gingivalis* are summarized in **Table 2**.

**Table 2:** Small molecules that inhibit *P. gingivalis*.

Small molecules	Chemical structure	Mechanisms	Antimicrobial activity	Reference
Synthetic molecules				
DANA	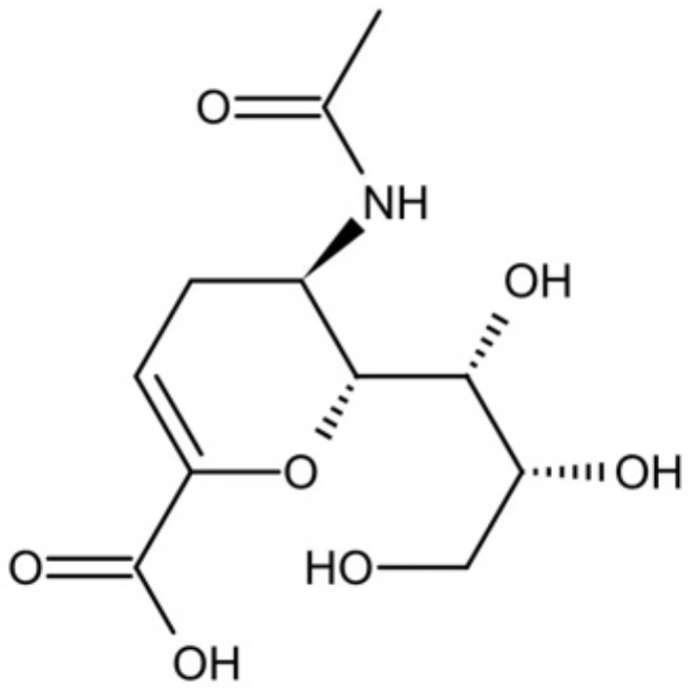	Inhibit growth and biofilm formation. Reduce expression of the *fimA*, *fimR*, and *fimS* genes and decrease gingipains activity. Inhibit TNF-α, IL-1β, and iNOS production in LPS-stimulated macrophages and prevent alveolar bone absorption and inhibited TNF-α and IL-1β production *in vivo*.	Inhibit *P. gingivalis* growth and biofilm formation at 1 mM	(Yu et al., 2021)
N7	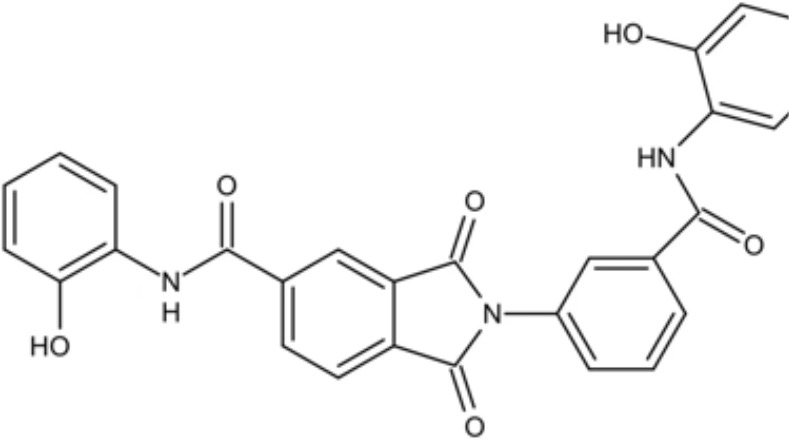	Inhibit *P. gingivalis* adherence to streptococci and reduced *its* virulence		(Roky et al., 2020)
N17	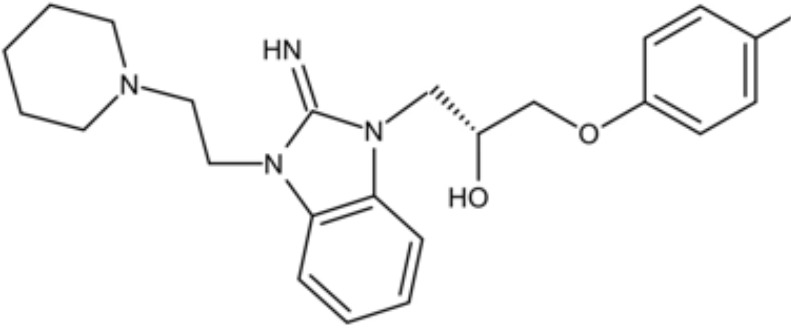			
V8	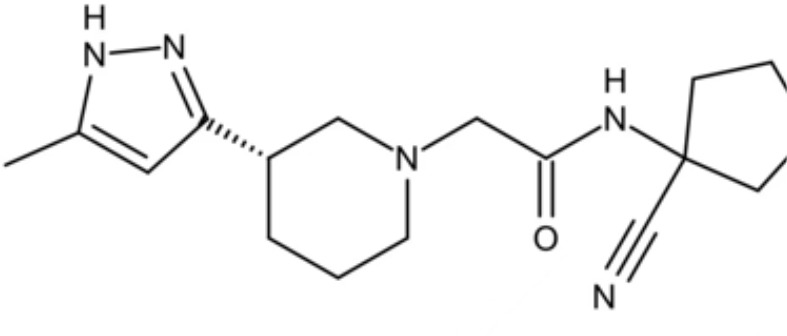			
PCP-III-201	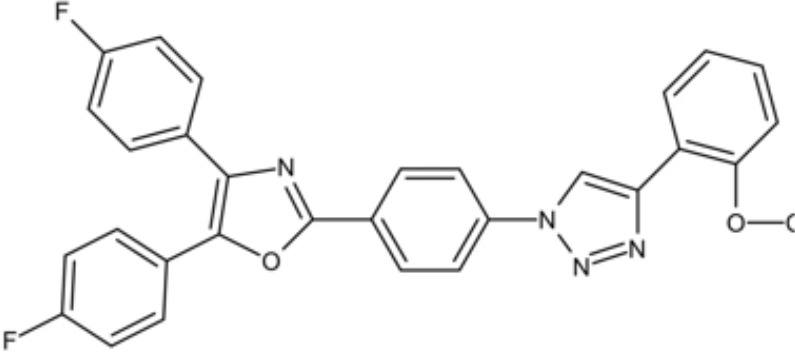	Inhibit the adherence of *P. gingivalis* to streptococci by interfering Mfa and antigenI/II interaction, and disrupt the formation of mixed biofilms	Inhibit 50% the incorporation of *P. gingivalis* into the three-species biofilm at 15 μM	(Tan et al., 2018)
			Inhibit preformed three-species biofilm in a dose-dependent way.	
1,2,3-triazole-based peptidomimetics		Inhibit *P. gingivalis* adherence to S. gordonii by inhibiting the interaction of antigen I/II and Mfa proteins		(Patil et al., 2016; Patil et al., 2019)
2A4	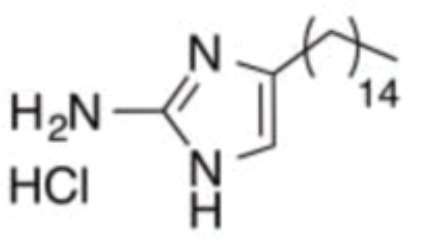	Inhibit *P. gingivalis*, and downregulate Mfa1 and fimA gene expression	MIC_90_: 20 μM	(Wright et al., 2014)
2D11	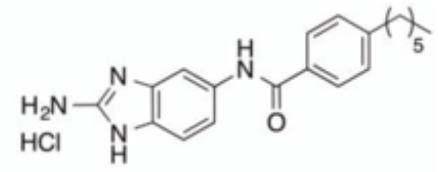		MIC50: 4.73 μM± 1.77	
2E11	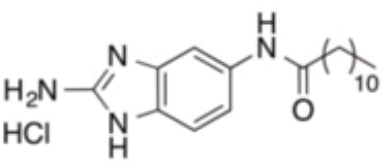		MIC50: 6.88 μM± 1.45	
7c	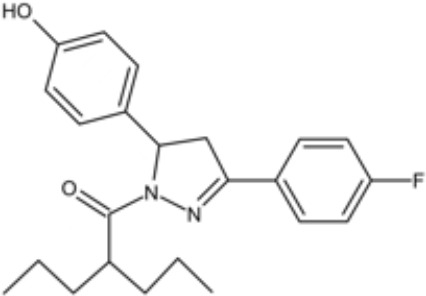	Inhibit microorganisms responsible for periodontitis including *P. gingivalis*, and exhibit notable effects on reducing inflammation by inhibiting TNF-α, IL-1β and IL-6	MIC_90_: 0.05 μg/ml	(Dong et al., 2021)
Natural Compounds				
Quercetin	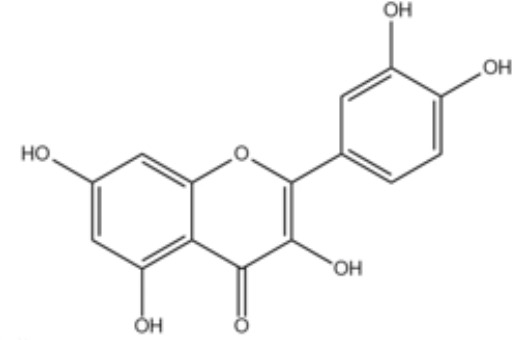	Inhibit gingipains activities and biofilm formation, and down-regulate the virulence-associated gene expressions of *P. gingivalis*	MIC_90_: 200 μM	(He et al., 2020)
			MBC_90_: 400 μM	
Quantum curcumin	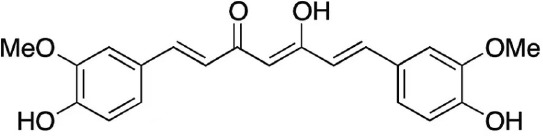	Inhibit planktonic cells and biofilms cells of *P. gingivalis*.In addition, inhibit gingipains.		(Singh et al., 2019)
Resveratrol	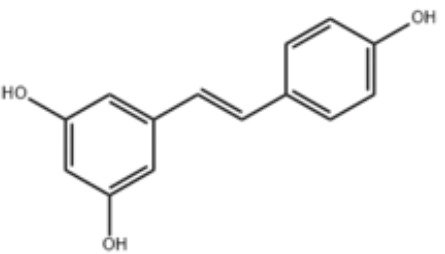	Inhibit the growth of *P. gingivalis* Reduce the *P. gingivalis* biofilm formation and its virulence by downregulating the expression of fimbriae and proteinases	MIC_90_: 156.25 μg/ml	(Kugaji et al., 2019)
			MBC_90_: 312.5 μg/ml	

MIC, minimum inhibitory concentration; MBC, minimum bactericidal concentration; MBIC, minimum biofilm inhibition concentration; MBRC, minimum biofilm reduction concentration.

### Candida albicans

*C. albicans* is an opportunistic pathogen in the oral cavity, which can cause oral fungal infections and increase the risk of oral epithelial carcinogenesis (Mayer et al., 2013). In addition, *C. albicans* robustly interacts with oral streptococci and can drive the microbial shift toward a more pathogenic microbiota that favors or aggravates the development of oral infectious diseases such as dental caries and periodontitis (Hajishengallis and Lamont, 2012).

Azoles, especially fluconazole (FLC) are the frontline treatment against fungal infections. However, with increasing drug resistance to current azole antifungals, small molecules have become a promising source for the development of novel antifungals (Kett et al., 2021). Azole antifungals inhibit sterol 14α‐demethylase (CYP51), resulting in synthesis disorder of ergosterol thereby affecting cell membrane integrity. Synthetic small molecules targeting CYP51 or inhibiting the synthesis of ergosterol are common strategies to inhibit *C. albicans*. Two kinds of small molecular azole derivatives (i.e. short and extended derivatives), have shown good binding affinity to CYP51, and thus potently inhibit CYP51 activity and the growth of *C. albicans* (Binjubair et al., 2020). Drug repurposing is also a promising approach to the identification of antifungals against *C. albicans* (Ashburn and Thor, 2004). A novel 1,2,4-triazole-indole hybrid molecule, (2-(2,4-Dichlorophenyl)-3-(1*H*-indol-1-yl)-1-(1,2,4-1*H*-triazol-1-yl)propan-2-ol,namely 8g, showed a broad-spectrum activity against *Candida* with low cytotoxicity, probably due to its inhibitory effects on ergosterol synthesis and phospholipase A2-like activity (Pagniez et al., 2020). Monika et al. synthesized a series of benzoxazole derivatives showing equivalent effects to commercially used azoles, which either interacted with exogenous ergosterol or blocked the synthesis of endogenous ergosterol. Among the library of 23 benzoxazoles featuring 2-mercaptobenzoxazole with the phenacyl moiety or respective alcohols, compound 5d showed good stability and water solubility, representing a good candidate in the treatment of andida infection (Staniszewska et al., 2021a).

*C. albicans* can form biofilms on the mucosal surface, which not only increase its virulence but also lead to drug resistance (Lohse et al., 2018). A study screened the Chembridge Small Molecule Diversity library containing 30,000 small molecules and identified 45 compounds which inhibited biofilm formation. Further investigation identified 4 compounds, namely CB06, CB14, CB36 and CB40, which inhibited biofilm formation and destroyed mature biofilm alone or in combination with other antifungals such as fluconazole and caspofungin (Lohse et al., 2020). Monika et al. screened 20 dibromobenzimidazole derivatives and identified a small molecule, namely 5h, which showed inhibitory effects on cell wall and reduced biofilm formation of *Candida albicans*. Besides, 5h was identified as a mitochondrial inhibitor of both *C. albicans* and *C. neoformans*, indicating its potential in the anti-fungal treatment (Staniszewska et al., 2021b).

Morphological transformation between hyphae and yeast phase in *C. albicans* is also relevant to its virulence and drug resistance (Vediyappan et al., 2013). A recent study repurposed 18 non-antifungal agents including antipsychotics, antiarrhythmics, proton pump inhibitors (PPIs), and identified 7 drugs that inhibited *C. albicans* in a dose-dependent manner, among which, chlorpromazine (CHL) and prochlorperazine (PCP) were fungicidal and the other 5 fungistatic. In addition, 3 candidate compounds, including chlorpromazine, prochlorperazine and drotaverine, inhibited germ tube formation (Kathwate et al., 2021). A high-throughput in silico study screened 584 compounds and identified 5 candidate molecules, namely U73122, disulfiram, BSK805, BIX01294, and GSKJ4, among which disulfiram showed excellent antifungal effects and inhibited 50% growth of *C. albicans* SC5314 strain at the concentration of 1mg/ml. Disulfiram also inhibited 50% growth of *C. albicans* biofilms at a concentration ranging from 32-128 mg/ml (Hao et al., 2021). A series of non-peptidic analogues of the broad-spectrum host defense peptides (HDPs) have also shown antifungal activities (Ryan et al., 2014). A compound 4 (C4) screened form HDP-mimic compound library can kill both the yeast and hyphal form of *C. albicans* at a concentration of 8 μg/ml (Menzel et al., 2017), suggesting a potential use in the control of invasive candidiasis.

*C. albicans* has several adhesins such as glutenin-like sequence (Als) family (Als1–Als7 and Als9), which mediate its adhesion to the host surface (Hoyer, 2001). Among Als family, the protein Als3 plays an important role in adhesion and hypha formation (Murciano et al., 2012). A small molecule, F2768-0318, shows inhibitory effects on the virulence factors related to adhesion and biofilm formation by inhibiting Als3 protein (Shinobu-Mesquita et al., 2020). Besides, machineries that modulate the stress responses are linked to fungal resistance. Heat shock protein 90 (Hsp90), an essential molecule in the stress responses of eukaryotes is also involved in the morphological transformation and drug resistance of *C. albicans* (Cowen and Lindquist, 2005; Taddei et al., 2014; Tiwari et al., 2015; Whitesell et al., 2019), and thus represents a promising target for the development of small molecules against Candidal infection. Yuan et al. screened 4 hsp90 inhibitors and identified a most potential compound, namely ganetspib, which exhibited significant synergistic activity with fluconazole in both planktonic cells and biofilms. The ganetspib in combination with FLC also down-regulated the expression of azole-targeting enzyme *ERG11* and efflux pump *CDR1, CDR2*, and *MDR1*. The synergistic effects of ganetspib with FLC *in vivo* also indicating its potential use as an antifungal enhancer of azoles in *Candida* infections (Yuan et al., 2021).

The over-expressed drug efflux pump on the cell membrane is another common virulence trait of *C. albicans* being related to azole resistance. Development of compounds that inhibit efflux pump and increase the concentration of drugs in the cell is a classic strategy to increase fluconazole sensitivity (Coste et al., 2004; Holmes et al., 2016). Kali et al. screened 2454 small molecules against *C. auris* and identified a *bis*-benzodioxolylindolinone CMLD012336, a 3,3-diarylated oxindole (also called azoffluxin), which showed synergistic interaction with fluconazole against a resistant strain of *C. auris*. Mechanistically, azoffluxin increased fluconazole sensitivity in both *C. auris* and *C. albicans* by inhibiting efflux pump CDR1 (Iyer et al., 2020). Another compound screened from a series of synthesized cyclobutene-dione (squarile) small molecules library, namely compound A, also showed inhibitory effect on MFS efflux pump CaMdr1p, and thus sensitized AD/CaMDR1 to FLC (Keniya et al., 2015). In addition, a small molecule ENOblock showed good inhibitory effects alone and in combination with FLC against *C. albicans* hypha and biofilm formation *in vivo*. Mechanistically, ENOblock interacted with *Ca*Eno1 and significantly inhibited the transglutaminase activity of CaEno1, and thus affected the growth and morphogenesis of *C. albicans* (Li et al., 2019). Small molecules that show inhibitory effects against *C. albicans* are summarized in **Table 3**.

**Table 3:** Small molecules that inhibit *C. albicans*.

Small molecules	Chemical structure	Mechanisms	Antimicrobial activity	Reference
Drug-repositioning				
8g	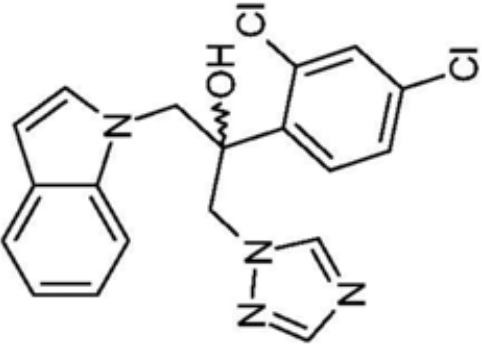	Show broad-spectrum activity against *Candida* with low cytotoxicity due to its inhibitory effects on ergosterol synthesis and phospholipase A2-like activity	MIC_90_: 0.5 μg/ml	(Pagniez et al., 2020)
			Inhibit ergosterol production by 82% and induced production of 14a-methyl sterols at 4μg/ml	
5d	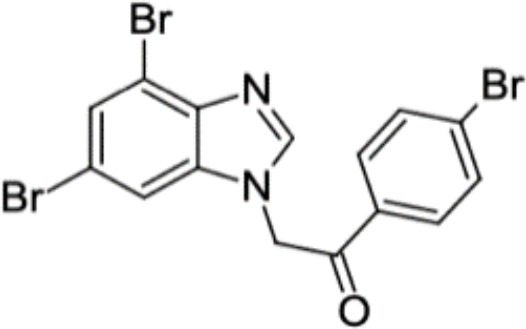	Interact with exogenous ergosterol as well as block the synthesis of endogenous ergosterol.	MIC_90_: 16 μg/ml	(Staniszewska et al., 2021a)
Phenotypic screening from molecule libraries				
azoffluxin	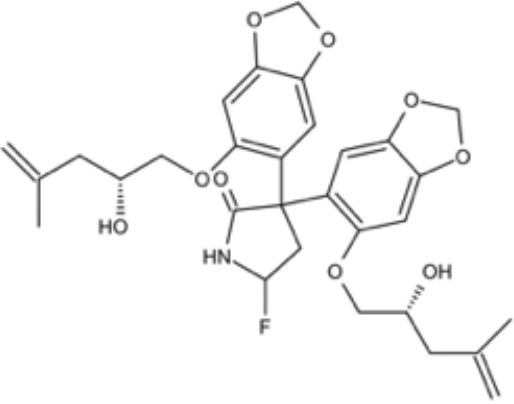	Increase fluconazole sensitivity in both *C. auris* and *C. albicans* by inhibiting efflux pump CDR1		(Iyer et al., 2020)
Compound A	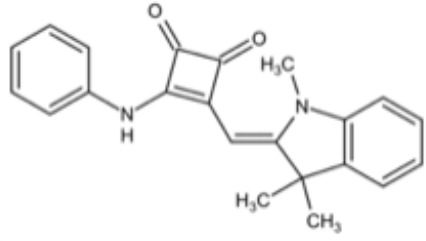	Inhibit MFS efflux pump CaMdr1p	FICI<0.05	(Keniya et al., 2015)
CB06	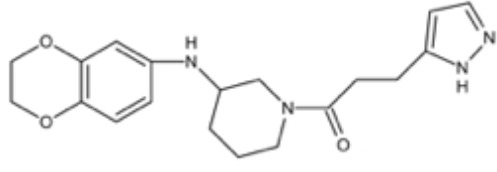	Inhibit biofilm formation and destroy mature biofilm in combination with other antifungals	Inhibit biofilm formation in the presence of FLC at 12.5μM	(Lohse et al., 2020)
CB14	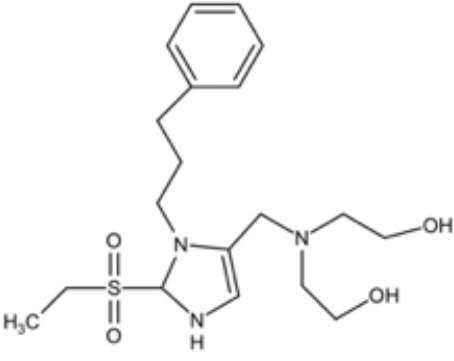		Destroy mature biofilm in the presence of caspofungi at 12.5μM	
CB36	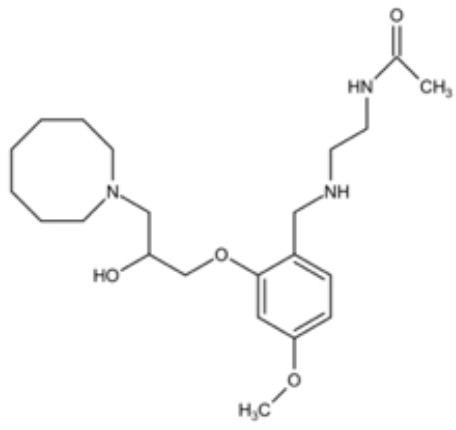		Inhibit biofilm formation and destroy mature biofilm in the presence of caspofungi at 12.5μM	
CB40	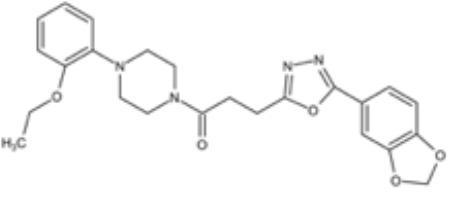		Destroy mature biofilm in the presence of caspofungi at 12.5μM	
C4	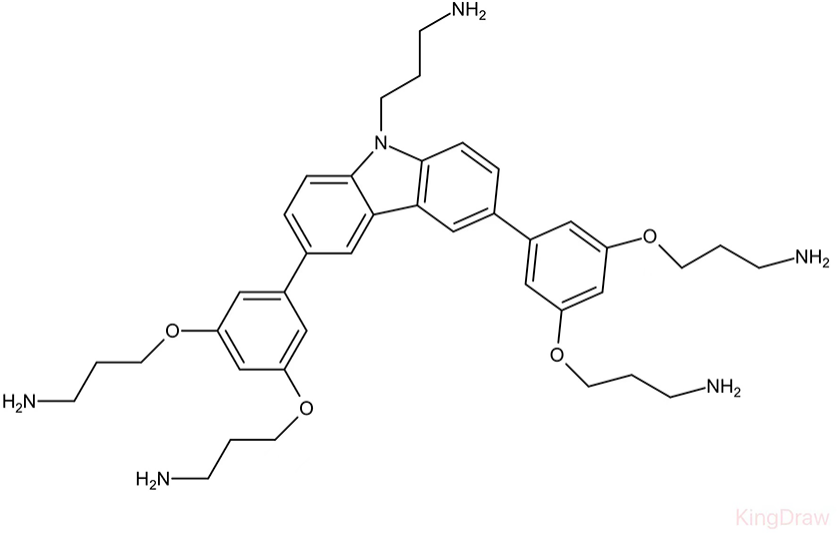	Kill both the yeast and hyphal form of *C. albicans*	MIC_90_: 2 μg/ml	(Menzel et al., 2017)
			MFC_90_: 8 μg/ml	
Disulfiram	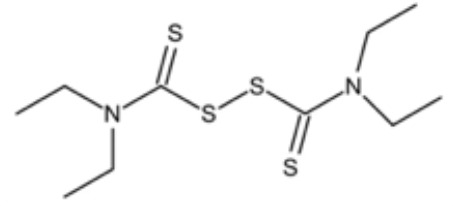	Inhibit *C. albicans* planktonic cells and biofilms	MIC_50_: 1 mg/ml	(Hao et al., 2021)
			sMIC_50_: 32-128 mg/ml	
ENOblock	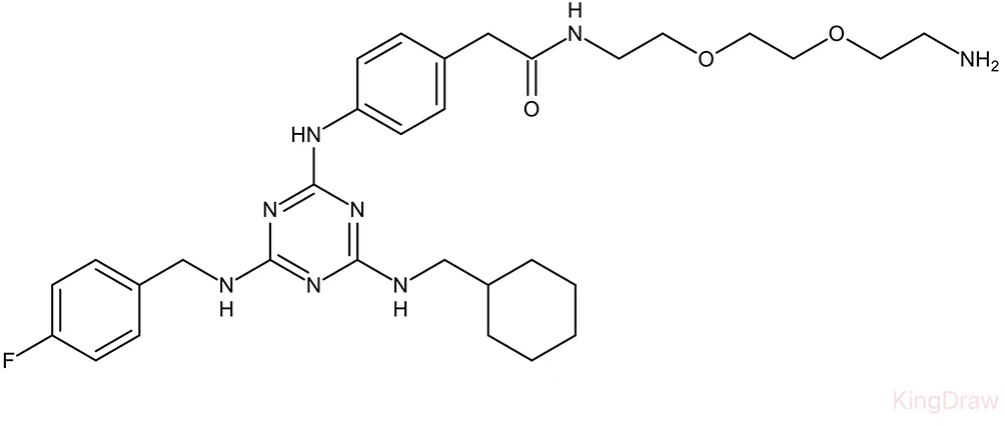	Show effects alone or in combination with FLC against *C. albicans* hypha and biofilm formation *in vivo*.	MIC_90_: 32 μg/ml	(Li et al., 2019)
		Interact with *Ca*Eno1 and inhibit the transglutaminase activity of CaEno1 in *C. albicans*	FICI<0.5 (*C.albicans* 0304103)	
F2768-0318	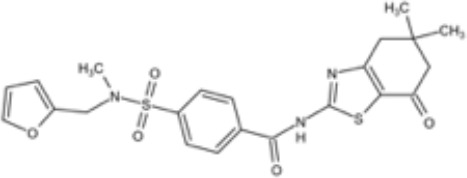	Inhibit *virulence factors* related to adhesion and biofilm formation by inhibiting Als3 protein	MIC_90_: 256 μg/ml	(Shinobu-Mesquita et al., 2020)
			MFC_90_: 256 μg/ml	
Ganetespib	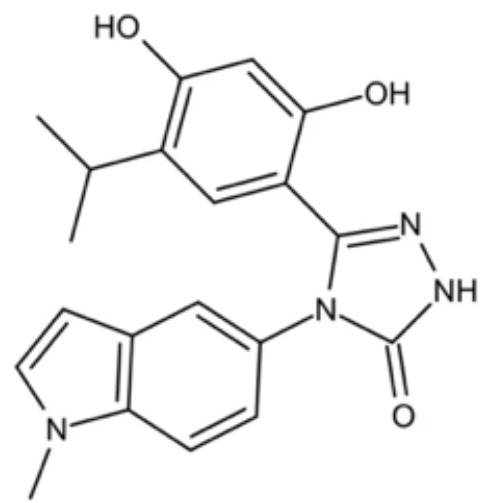	Show synergistic activity with fluconazole in both planktonic cells and biofilms, and down-regulate the expression of azole-targeting enzyme gene *ERG11* and efflux pump genes *CDR1, CDR2*, and *MDR1*	FICI<0.05	(Yuan et al., 2021)
5h	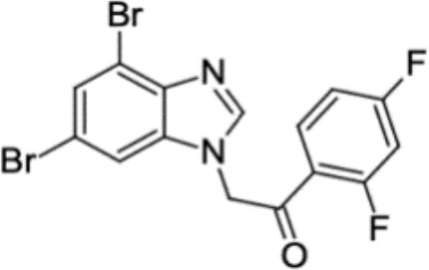	Inhibit cell wall and inhibit biofilm formation.	MIC_50_: 8 μg/ml	(Staniszewska et al., 2021b)
		Inhibit mitochondrion in both *C. albicans* ref and *C. neoformans*		

MIC, minimum inhibitory concentrations; MFC, minimum fungicidal concentrations; sMIC, sessile minimum inhibitory concentrations; FICI, fractional inhibitory concentration index.

## Small Molecules That Inhibit Quorum Sensing System

Different microorganisms exist in oral microbiota, and the robust microbial interactions have close relationship with host health and diseases. Small molecules which disrupt microorganism interactions may rescue the microbial disequilibrium, contributing to the management of oral infectious diseases. Quorum sensing (QS) system, a mechanism of microbial interaction, involves signaling molecules that enable a cell to sense cell density (Padder et al., 2018). It relies on the production, release and recognition of self-induced signal molecules and regulates various biological behaviors including biofilm formation, microbial virulence and resistance (Papenfort and Bassler, 2016). Small signal molecules concentrated in the extracellular environment mediate QS in fungi. The mechanisms accountable for the accumulation of these molecules include passive diffusion, efflux pumps and specific transporters (Padder et al., 2018).

ComA is a critical component of *S. mutans* QS system (Havarstein et al., 1995). Ishii et al. found a compound that inhibited the peptidase domain (PEP) of ComA, thus disrupting the QS system and inhibiting biofilm formation of *S. mutans*. This compound also inhibited the PEP of *Streptococcus pneumoniae* and *Streptococcus. oralis* (Ishii et al., 2017). Kaur et al. synthesized a compound called 1,3-disubstituted urea derivatives, which inhibited the activity of ComA by binding to the active sites of PEP. 1,3-disubstituted urea derivatives was able to inhibit the formation of *S. mutans* biofilm alone or in combination with low concentration fluoride (Kaur et al., 2016). DMTU (1,3-di-m-tolyl-urea) is an aromatic compound showing antibiofilm effects against *Streptococcus mutans* by inhibiting its quorum sensing pathway (comDE) (Kaur et al., 2017). In addition, DMTU significantly inhibited the formation of multispecies biofilms consisting of *Streptococcus gordonii*, *Fusobacterium nucleatum*, *Porphyromonas gingivalis* and *Aggregatibacter actinomycetemcomitans* at 12.5μM without affecting cell viability. DMTU also down-regulated the expression of virulence genes in *P. gingivalis* such as mfa1, rgpA and rgpB (Kalimuthu et al., 2020).

Autoinducier-2 (AI-2) is anthor QS molecule that is produced and recognized by *S. mutans*, *S. gordonii*, *P*. *gingivalis* and *Fusobacterium nucleatum* (Shao and Demuth, 2010). Park et al. (Park JS et al., 2017) found that 3-(dibromomethylene) isobenzofuran-1(3H)-one derivatives inhibited the activity of AI-2 in *F. nucleatum* and reduced its biofilm formation. Another study found that D-galactose not only inhibited AI-2 activity, but also dose-dependently inhibited the biofilm formation of *F. nucleatum*, *P. gingivalis*, and *T. forsythia* (Ryu et al., 2016).

## Novel Drug Delivery Systems of Antimicrobial Small Molecules

Drug delivery system (DDS) is an advanced means that improves the therapeutic characteristics of conventional drugs over the last few decades. Various pharmacological properties of drugs, such as pharmacokinetics and biodistribution can be altered by using small-scale DDS such as nanoparticles (NP) and microparticles. Currently, nanotechnology has been extensively explored in DDS concerning the antimicrobial properties of drugs. The incorporation of drugs into nanoparticles and nanocomposites by physical encapsulation, adsorption, or chemical conjugation, can notably enhance the pharmacokinetics and therapeutic efficacy of the drugs in the treatment of infectious diseases as compared to their free counterparts (Zhang et al., 2010). In recent years, nano-based delivery system containing small molecular antimicrobials, either derived from natural or synthetic compounds, has attracted increasing attentions in the field of oral microbial infection control. Nowadays, since antimicrobial resistance (AMR) has been arousing global concerns in the clinical practice, a variety of innovative nanotechnologies have been applied to improve the therapeutic properties of small molecules. Antimicrobial small molecules can be conjugated with or encapsulated into various nanoparticles including organic polymers and metallic nanoparticles (MNPs), exhibiting elevated solubility (Yang et al., 2009; Nicolosi et al., 2015), biocompatibility (Wang et al., 2020) and controlled release (Barman et al., 2014), and thus benefit the control of oral infectious diseases.

### Organic Polymers

#### Polysaccharides

Polysaccharides are notable for targeted drug delivery systems as natural biomaterials. Polysaccharides are inexpensive and possess promising biocompatibility and biodegradability. In the polysaccharides-based drug delivery system, the loaded agents can be absorbed into external compartments or limited within the external surface, which can augment the stability and aqueous solubility of drugs (Barclay et al., 2019). Maghsoudi et al. compared the anticariogenic activities of three curcumin-loaded polysaccharide nanoparticles including starch, alginate and chitosan. The results showed that polysaccharide nanoparticles possessed enhanced antimicrobial activities as compared to pure curcumin. Among the three curcumin-loaded polysaccharide nanoparticles, the chitosan nanoparticles showed the largest amount of release and the best anticariogenic properties particularly at lower pH (Maghsoudi et al., 2017). Jahanizadeh et al. incorporated carboxymethyl starch (CMS), a modified starch with unique negatively charged groups with chitosan to develop a novel curcumin-loaded nanocomposite. This nanocomposite showed enhanced antimicrobial and antibiofilm properties against *S. mutans*. Meanwhile, this nanocomposite had a smaller nanoscale with an average size of 35.9 nm and exhibited excellent curcumin entrapment efficiency of 91% (Jahanizadeh et al., 2017).

#### Poly Lactic-Co-Glycolic Acid Copolymer

Poly lactic-co-glycolic acid (PLGA) is a synthetic polymer extensively utilized in the field of drug delivery system due to its biocompatible, biodegradable and sustained-release characteristics (Kapoor et al., 2015). Numerous PLGA-based drug delivery systems have been designed with different hydrophilic moieties such as poly ethylene glycol (PEG) or poly ethylene oxide (PEO) to increase the solubility of small molecules (Mir et al., 2017). Gürsu et al. synthesized farnesol-loaded PLGA nanoparticles to enhance the bioavailability of farnesol. PLGA chemically interacted with the OH group of farnesols, and 22.5% of the equivalent amounts of nanoparticles achieved a similar inhibitory effect against *C. albicans* (Gürsu, 2020). Ahmadi et al. modified a photoexcited orthodontic adhesive by incorporating curcumin-loaded PLGA nanoparticles and explored its anti-biofilm effects against *S. mutans*, shear bond strength (SBS) and adhesive remnant index (ARI). The results showed that the antimicrobial activity of the blue laser-7% wt. Curcumin-PLGA nanoparticles was comparable to 2% chlorhexidine. Moreover, the modified adhesive showed the highest SBS value and comparable ARI relative to the original pure adhesive (Ahmadi et al., 2020).

### Liposome

Liposome owns many critical clinical features, especially excellent biocompatibility and capability of encapsulating both hydrophilic and hydrophobic compounds (Samad et al., 2007). The most attractive feature of liposome delivery systems is enhancing pharmacokinetic properties and bactericidal activities of the new or existing drugs as well as reducing their drugs’ adverse effects (Alhariri et al., 2013). Nicolosi et al. reported that fusidic acid-loaded fusogenic liposomes increased the antimicrobial activities and broadened the antimicrobial spectrum in contrast to free drugs.The fusogenic small unilamellar vesicles may elevate the penetration of lipophilic drug into microorganisms and thus improve its antimicrobial properties (Nicolosi et al., 2015). However, the nanocarrier without being activated by specific stimuli cannot specifically reach and work in the targeting sites *in vivo*. Controlled drug release is promising for the trigger-release of liposomes in the clinical applications (Bibi et al., 2012). Mizukami et al. developed two enzyme-activity-triggered drug release systems by combining an antimicrobial peptide temporin L (TL) with surface-anionic liposomes. Protease and phosphatase were chosen as the target enzymes. For the protease-triggered system, a branched peptide that suppressed membrane-damaging activity was fabricated by modifying the cationic Lys residue of TL. As for the phosphatase-triggered system, a neutral amino acid with an anionic phosphorylated amino acid in the lipophilic region of TL was replaced. The phosphopeptides alleviated its membrane-damaging activity so that controlled release was achieved (Mizukami et al., 2017).

### Metallic Nanoparticles

Metallic nanoparticles can efficiently cope with resistant microbial strains due to its potential antimicrobial activity (Rai et al., 2017). A vast number of studies have demonstrated that silver nanoparticles have strong antimicrobial activities against various microorganisms including resistant pathogens (Rai et al., 2017). However, the toxicity of silver and many other metallic nanoparticles limits their clinical application (Mizukami et al., 2017). Yin et al. developed silver nanoparticles containing EGCG as reducing agent, which showed elevated biocompatibility than silver nitrate (AgNO3). Moreover, it also exhibited much lower MIC and MBC against *S. mutans* and potently inhibited acid and polysaccharide production (Yin et al., 2019). In contrast to other NPs, gold NPs (Au NPs) have many advantages, including controllable synthesis, versatility in surface modification, and admirable biocompatibility (Zhao et al., 2013; Yang et al., 2017). Although gold NPs usually show weak inherent antimicrobial activities (Rai et al., 2017), they can be modified or coated on different surfaces to function with antimicrobial properties. Wang, et al. synthesized a series of N-heterocyclic molecule-coated gold NPs, among which 2-mercaptoimidazole (MI)- and 3-amino-1,2,4-triazole-5- thiol (ATT)-capped Au NPs showed broad-spectrum antibiofilm activities against MDR bacteria, including MRSA and MDR *Escherichia coli* (MDR *E. coli*). These NPs directly contacted and disrupted the cell wall of microorganisms, thus less likely to induce antimicrobial resistance. Moreover, taking into account the advantages of ultrasound-assisted coating method being highly durable and able to withstand a large number of washing cycles, Wang et al. used sonochemistry to coat Au NPs on the surface of fabrics, which showed excellent antimicrobial activity (Wang et al., 2020). S. Jabir et al. also reported that a linalool loaded on glutathione-modified gold nanoparticles in spherical shape was antimicrobial against gram-positive bacteria representing a biocompatible and less complicated and time-consuming approach to the delivery of antimicrobial small molecules (Jabir et al., 2018). Lu et al. developed a small molecule 2-mercapto-1-methylimidazole-capped Chitosan-functionalized nanocomposites whose cationic amine rendered transport of the nanocomposites towards the negatively charged bacterial cell surface. With the alliance between small Imidazol molecules and gold nanoparticles, the nanocomposites exhibited bactericidal and biofilm disruption effects against both Gram-negative *E. coli* and Gram-positive *S. aureus*. Besides, these nanocomposites can be easily synthesized with low toxicity, showing good potential in clinical applications (Jabir et al., 2018). Apart from incorporating with inherent antimicrobial small molecules, Zhao et al. incorporated a non-antimicrobial molecule 4,6-diamino-2-pyrimidinethiol (DAPT) with Au-NPs. The DAPT-decorated Au-NPs was able to inhibit most of MDR Gram-negative bacteria such as *E. coli* and MDR *Pseudomonas aeruginosa* by suppressing energy metabolism and damaging bacterial membrane (Zhao et al., 2010).

### Tetrahedral Framework Nucleic Acids

In recent years, tetrahedral framework nucleic acids (tFNAs) have attracted increasing attention due to their editability and biocompatibility (Zhang et al., 2020). Drug delivery based tFNAs has been proposed and investigated broadly over the past years (Li et al., 2021; Zhang et al., 2022). Zhang et al. synthesized tFNA/His-5, and by the modification of tFNAs, His-5 showed increased transport efficiency and improved anti-fungal effect (Zhang B et al., 2021). Moreover, Zhang et al. also developed tFNAs with a controllable conformation as a delivery vehicle for antisense oligonucleotides, which provided a better platform for the applications of antisense antibacterial therapeutics (Zhang et al., 2018). Besides, Sun et al. found tFNAs-ampicillin had a better antimicrobial effect and lower levels of drug resistance development than free ampicillin (Sun et al., 2020). However, it is doubtful that tFNAs could carry some molecules that are beyond their size and weight. Besides, attaching nucleic acids with complex secondary structures may inhibit the uptake process. Wider potential to deliver different cargos still need to be explored (Zhang et al., 2020).

### Virus-Derived Nanoparticles

Although metallic nanoparticles have been explored as nanocarriers for small molecular antimicrobials, its accumulation within human body and interactions with organs and microenvironment have aroused concerns for its potential toxicity. Metallic nanoparticles can also stimulate the expression of cytokines that generate cytotoxicity, immunotoxicity, and genotoxicity (Zazo et al., 2017). To avoid these side effects, organic nanoparticles have attracted increasing attentions. Currently, virus-derived nanoparticles (VNPs), a self-assembly-competent protein have shown advantages such as biodegradable, cost-effective and highly modifiable (Verma et al., 2018). Velázquez-Lam et al. conjugated EGCG to turnip mosaic virus (TuMV) particles, and the EGCG-TuMV VNPs not only maintained TuMV structure but also exhibited elevated antimicrobial and antibiofilm activities compared with free EGCG. Moreover, EGCG-VNP showed a high stability after six months of being stored at 4°C. Of note, the various modifiable sites of VNPs provide the versatility for novel nanocarriers in drug delivery system (Velaäzquez-Lam et al., 2020).

## Conclusion

Oral microbial dysbiosis is the most essential causative factor for common oral infectious diseases including dental caries and periodontal diseases. Small molecules with potent antimicrobial activity, high selectivity, and low toxicity are promising for the ecological management of oral diseases. Many small molecules have already been repurposed, screened and designed. However, many issues have yet to be solved. Although antimicrobial activities have been revealed for many small molecules, their mode of action and underlying mechanism are still unclear. The exact targets of some novel small molecules have yet to be identified. Therefore, structural modifications and optimization based on the candidates with confirmed mechanism and targets are promising. In addition, although the antimicrobial activity of small molecules against the keystone pathogens such as *S. mutans* and *P. gingivalis* has been demonstrated, their ecological impact on the disease-provoking microbiota still needs further investigation in a more sophisticated microbial consortium and validation *in vivo*. Small molecules that disrupt microbial interactions without necessarily killing the bacteria is also promising, particularly in the light of robust advancement in the development of drug delivery system that enhances its substantivity and specificity. Finally, comprehensive evaluation of the long-term cytotoxicity *in vivo* is still needed to better translate those promising candidates to the clinical application.

## Author Contributions

RY and XX conceived and designed the review. SY, XL, JZ, and YS wrote the first draft of the manuscript. RY and XX helped revise the manuscript. All authors contributed to the article and approved the submitted version.

## Funding

This study was supported by the National Natural Science Foundation of China (81771099, 81800989).

## Conflict of Interest

The authors declare that the research was conducted in the absence of any commercial or financial relationships that could be construed as a potential conflict of interest.

## Publisher’s Note

All claims expressed in this article are solely those of the authors and do not necessarily represent those of their affiliated organizations, or those of the publisher, the editors and the reviewers. Any product that may be evaluated in this article, or claim that may be made by its manufacturer, is not guaranteed or endorsed by the publisher.
